# Rational design of fluorescent probe for Hg^2+^ by changing the chemical bond type†

**DOI:** 10.1039/c8ra00295a

**Published:** 2018-03-29

**Authors:** Tengli Cui, Shengzhen Yu, Zejing Chen, Rui Liao, Xinglin Zhang, Qiang Zhao, Huibin Sun, Wei Huang

**Affiliations:** China Key Laboratory of Flexible Electronics (KLOFE) & Institute of Advanced Materials (IAM), Jiangsu National Synergistic Innovation Center for Advanced Materials (SICAM), Nanjing Tech University (NanjingTech) 30 South Puzhu Road Nanjing 211816 P. R. China iamhbsun@njtech.edu.cn iamwhuang@njtech.edu.cn; Shaanxi Institute of Flexible Electronics (SIFE), Northwestern Polytechnical University (NPU) 127 West Youyi Road Xi'an 710072 China; Key Laboratory for Organic Electronics and Information Displays & Institute of Advanced Materials (IAM), SICAM, Nanjing University of Posts & Telecommunications 9 Wenyuan Road Nanjing 210023 P. R. China

## Abstract

Two kinds of fluorescent probes DFBT and DFABT, and their corresponding water-soluble compounds WDFBT and WDFABT, based on the trimers containing a benzo[2,1,3]thiadiazole moiety and two fluorene moieties are synthesized. Their luminescent behavior towards Hg^2+^ ions and other various metal ions in organic and water solutions are studied in detail *via* absorption and emission spectroscopy. All these probes show a selective “on–off-type” fluorescent response to Hg^2+^ ions in solution over other metal ions with a maximum detection limit of 10^−7^ M. Importantly, the probe type can be changed from irreversible to reversible by altering the bridge mode between the functional units from C≡C triple bond to C–C single bond. Their detection mechanisms towards Hg^2+^ are studied in detail *via* mass spectrometry and Job plots, which are attributed to irreversible chemical reaction for DFABT and WDFABT and a reversible coordination reaction for DFBT and WDFBT respectively. Our research results about this kind of organic fluorescent probe provide valuable information to the future design of practical Hg^2+^ fluorescent probes.

## Abstract 2

Two kinds of fluorescent probes for Hg^2+^ with different detection mechanism have been realized by simply changing the chemical bond.

## Graphical abstract

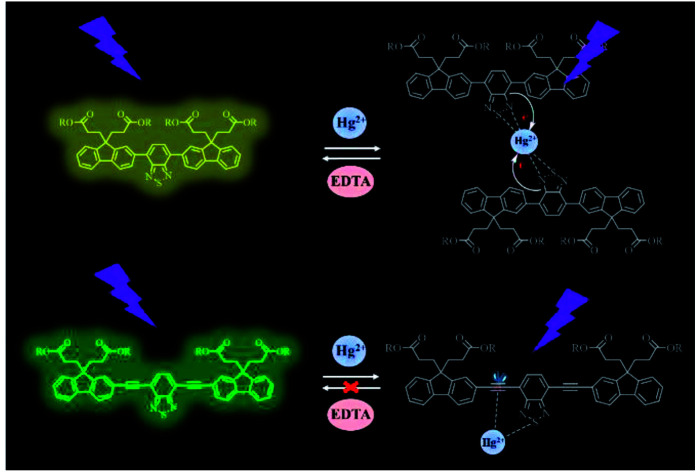

## Introduction

Mercury is well-known as an extremely hazardous chemical in biology and environment, and is especially toxic for mammals.^1–4^ It can enter the body through the skin, digestive system or respiratory system directly.^5,6^ Moreover, it can accumulate in the environment and enter the human body eventually through the food chain by its high accumulation ability, which causes serious damage to the central nervous, DNA, mitosis and endocrine systems.^7–9^ According to the World Health Organization, the maximum acceptable level of mercury ion in drinking water is 1 μg L^−1^.^10^ Consequently, the detection and quantification of Hg^2+^ ions, especially in the real aqueous environment, is of the utmost importance.

Fluorescent chemosensors as one of the most effective detection methods of mercury ions possess the advantages of simplicity, short responsive time, excellent selectivity and sensitivity, which have become a powerful tool for sensing trace amounts of Hg^2+^.^11–14^ In the last decades, many organic fluorescent probe molecules with different photo-active moieties such as 1,8-napthalimide, coumarin, pyrene, anthracene, BODIPY, squaraine, xanthanes, cyanine, rhodamine, and fluorescein, have been reported.^15^ However, these sensor systems are difficult to be applied widely because of their poor chemical stability, water-solubility and reversibility, which greatly restrict their applications *in vitro* and *in vivo*.^2,16–21^ And, to date, the design and development of fluorescent chemosensors with excellent performance still is a challenge.

Benzo[2,1,3]thiadiazole (BT) moiety is a kind of well-known metal ions receptor fluorophore with widespread application as fluorescent probe due to its valuable characteristics, such as simple, stable and malleable chemical structure, large Stokes shift in the visible spectral region, high fluorescence quantum yields, and better mercury ion coordination selectivity.^22–25^ However, to the best of our knowledge, the reported fluorescent probes based on BT for real-time detection of Hg^2+^ in pure aqueous solution are still relatively rare, especially for the reversible one, which is important *in vitro* and *in vivo* application.^21^ Thus, the design and development of effective fluorescent probe of Hg^2+^ with excellent water-solubility, high sensitivity and selectivity, especially with reversibility based on BT moiety is of great necessary.

**Scheme 1:**
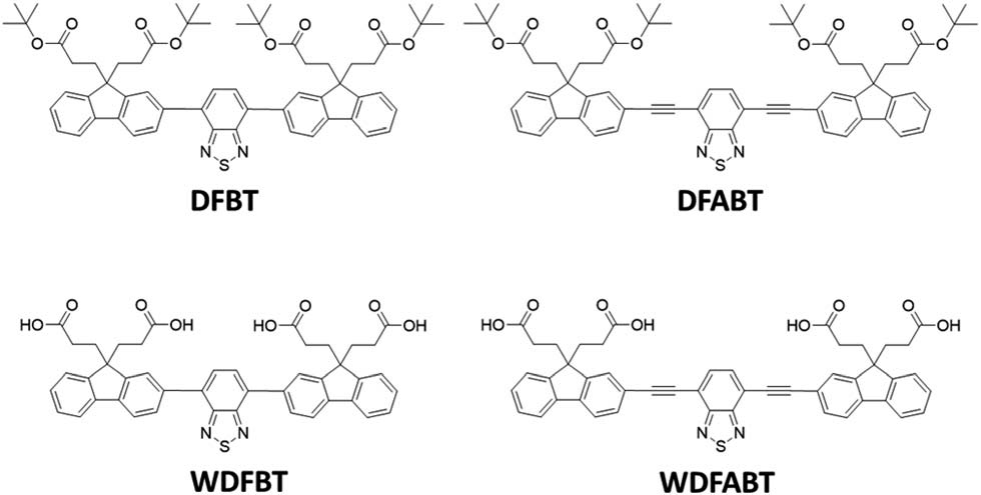
Chemical structure of DFBT, DFABT, WDFBT and WDFABT.

In this contribution, we designed two π-conjugated donor–acceptor–donor type trimers DFBT and DFABT and their corresponding water-soluble compounds WDFBT and WDFABT, based on fluorene moiety due to its great electron-donating ability,^26–29^ of which contained BT as the receptor for Hg^2+^ considering the specific interaction between Hg^2+^ and the sulfur or nitrogen atom in BT unit (see in Scheme 1). Meanwhile, carboxyl groups were introduced to improve the water-solubility and realized the detection of Hg^2+^ in pure aqueous solution. Besides, different bridged mode, such as single and triple bond, between two functional units were chose to manipulate the sensing performances of the probe for Hg^2+^. Based on above design ideas, the sensing properties including selectivity, sensitivity, and reversibility of the two Hg^2+^ ions fluorescent probes were investigated in detail.

## Experimental section

### Materials and methods

2-Bromofluorene, 2,7-dibromofluorene, tetrabutylammonium bromide, bispinacolatodiboronmin, Pd(dppf)_2_Cl_2_, 4,7-dibromo-2,1,3-benzothiadiazole, trifluoroacetic acid, trimethylsilylacetylene, Hg(ClO_4_)_2_, nitrate salts of metal ions (Ba^2+^, Ag^+^, Ca^2+^, Co^2+^, Cr^3+^, Cu^2+^, K^+^, Na^+^, Mg^2+^, Al^3+^, Ni^2+^, Zn^2+^), ethylene-diaminete-traacetic acid disodium salt and all other chemicals were purchased from Shanghai J&K company. The target molecules were synthesized according to the reported references, and the detailed synthetic routes are shown in ESI.†^22,30^ All the solvents and metal salts were used as received.

^1^H NMR (300 MHz, 500 MHz) and ^13^C NMR (100 MHz) spectra were recorded at room temperature on Bruker Ultra shield Plus 300 and 500 Hz instrument using CDCl_3_ and (CD_3_)_2_SO as the solvent and TMS as the internal standard. The mass spectra were measured on a Bruker autoflex MALDI-TOF/TOF mass spectrometer. The column chromatography was carried out using silica gel (200–300 mesh). UV-vis absorption spectra were measured by employing a Shimadzu UV-3600 UV-VIS-NIR spectrophotometer. Photoluminescent spectra were recorded on a FL-400PC spectra spectrophotometer. The pH values were measured on a Mettler-Toledo PHS-3E pH meter.

Spectrophotometric titrations for UV-vis absorption and PL spectra were performed on 10 μM solutions of DFBT and DFABT in CH_2_Cl_2_ and WDFBT and WDFABT in the buffer aqueous solution of citric acid and Na_2_HPO_4_, 2 mL solution was added into quartz cuvette, then the UV-vis absorption and PL spectra of samples were recorded respectively when the aliquots of fresh Hg^2+^ in deionized water were added. Other twelve kinds of metal ions (Ba^2+^, Ag^+^, Ca^2+^, Co^2+^, Cr^3+^, Cu^2+^, K^+^, Na^+^, Mg^2+^, Al^3+^, Ni^2+^, Zn^2+^) were also researched in same methods to study the selectivity of the probes.

## Results and discussion

### General synthetic procedure

The detailed synthetic routes of DFBT, WDFBT, DFABT and WDFABT are described in the ESI.† The structure of the two dyes were confirmed by ^1^H NMR, ^13^C NMR and MALDI-TOF analyses (Fig. S1–S14†). DFBT and DFABT were prepared by the palladium-catalysed Suzuki and Sonogashira reaction respectively.^22,30^ And then, WDFBT and WDFABT were obtained by removing the *t*-butyl ester group under a strong acid condition at 0 °C. The conjugacy was regulated by changing the coupling way between the fluorine and BT moiety, and then the binding ability to Hg^2+^ of the probes was regulated.

### Spectroscopic properties

**Fig. 1:**
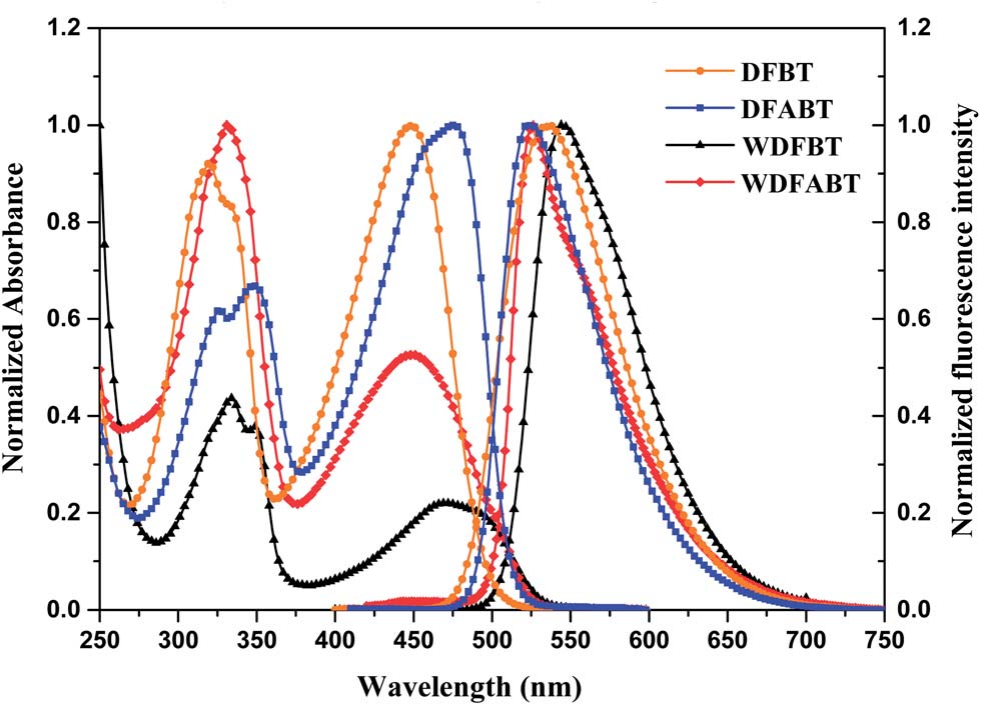
Normalized absorption and emission spectra of DFBT and DFABT in CH_2_Cl_2_ (10^−5^ M), WDFBT and WDFABT in buffer solution (10^−5^ M).

The absorption and emission spectra of DFBT and DFABT in CH_2_Cl_2_ solution, WDFBT and WDFABT in buffer solution of citric acid and Na_2_HPO_4_ were measured. As shown in Fig. 1, the absorption spectra of all the dyes displayed typical characteristic peak of fluorene and BT moiety, with an intense bands around 330 nm and a weak band around 460 nm, corresponding to S_0_ → S_1_ (π–π*) transitions of fluorene moiety and intramolecular charge transfer (ICT) state between donor (fluorene) and acceptor (BT) in the dyes respectively.^29,31^ All the dyes, except WDFABT, exhibited the intense emission at room temperature in solution when excited at their absorption maxima. And the photoluminescence spectra for all the dyes are broad and structureless, which consistent with ICT characteristics for such kind of dyes.^25^ Furthermore, the emission spectra of DFABT and WDFABT exhibited an obvious blue-shift with green color compared with that of DFBT and WDFBT, which probably due to the inhomogeneity in the conjugation path caused by the triple bond.^32^ The relative fluorescent quantum yields (*ϕ*) are 0.69, 0.58, 0.71 and 0.09 for DFBT, WDFBT, DFABT and WDFABT respectively, using rhodamine-B as a reference. And the low fluorescent quantum yield of WDFABT may be attributed to self-quenching because of bad solubility in water caused by the rigid structure.

### Absorption and fluorescence response for Hg^2+^

**Fig. 2:**
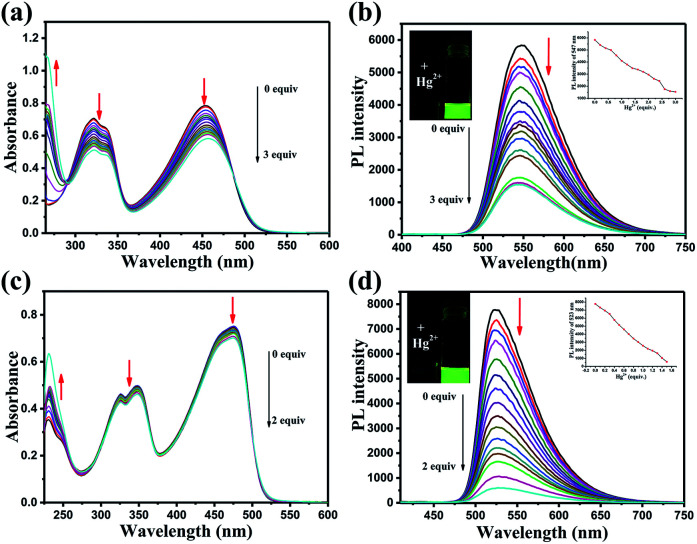
Changes in the absorption and fluorescence of DFBT and DFABT in CH_2_Cl_2_ solution with various amounts of Hg^2+^ ions: (a) and (c) UV-vis absorption spectra change of DFBT and DFABT in CH_2_Cl_2_ solution (10 μM), respectively; (b) and (d) fluorescence spectra change of DFBT and DFABT in CH_2_Cl_2_ solution (10 μM) excited at 360 nm, respectively. Inset: emission intensity change of DFBT and DFABT induced by different equiv. of Hg^2+^ ions, and fluorescent color change of DFBT and DFABT upon addition of Hg^2+^ ions.

The responses of DFBT and DFABT for Hg^2+^ were investigated by absorption and emission spectra. The absorption response towards Hg^2+^ ions in CH_2_Cl_2_ solution are shown in Fig. 2. With the continuous addition of Hg^2+^ ions, the absorption spectra of DFBT and DFABT decreased progressively accompanied by slight red-shift (see in Fig. 2(a) and (c)).

It was well known that fluorescent signal was more sensitive towards minor changes that impact the electronic properties of the molecular probe.^22^ Therefore, the response of DFBT and DFABT to Hg^2+^ ions were also studied by emission spectroscopy (see in Fig. 2(b) and (d)). Upon addition of Hg^2+^ ions, the intensity of the emission band of DFBT at 547 nm and DFABT at 525 nm gradually decreased. Hence, both DFBT and DFABT could be used as “on–off-type” fluorescent probe to Hg^2+^ ions (see in image of inset of Fig. 2(b) and (d)). Moreover, the fluorescence intensity of the two probes exhibited a better linear relationship with equivalent of Hg^2+^ ions (see in plot of inset of Fig. 2(b) and (d)). Compared with DFBT, however, the emission intensity of DFABT decreased faster, which indicated that DFABT served as a probe for mercury ions was more sensitive than DFBT. This may be due to the synergetic coordination effect of the triple bond, which enhances the binding ability between DFABT and Hg^2+^ ions.^33^

**Fig. 3:**
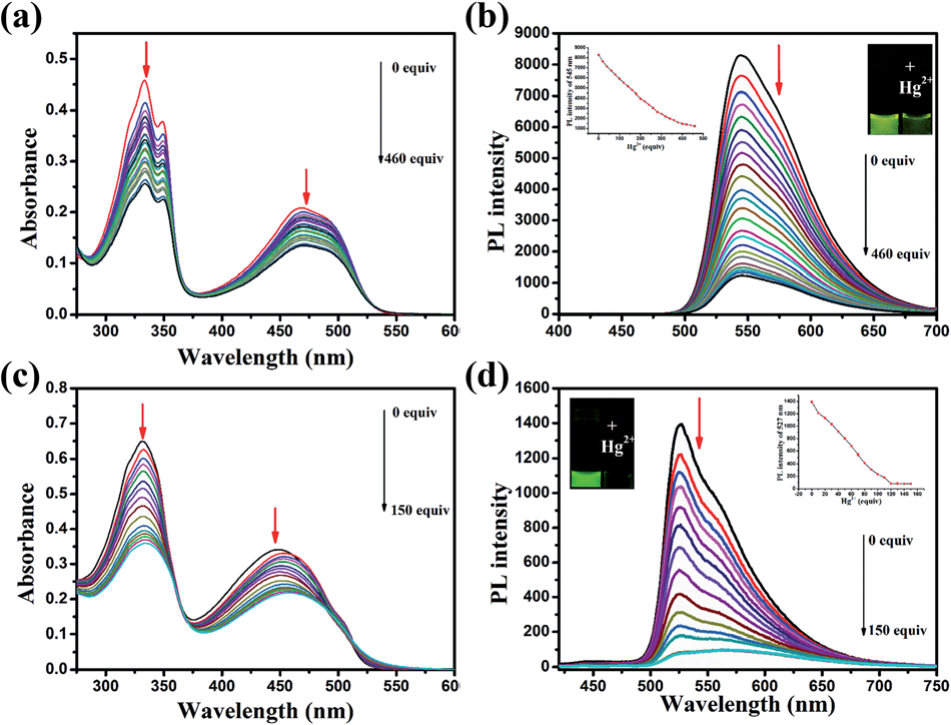
Changes in the absorption and fluorescence of WDFBT and WDFABT in buffer solution with various amounts of Hg^2+^ ions: (a) and (c) UV-vis absorption spectra change of WDFBT and WDFABT in buffer solution (10 μM), respectively; (b) and (d) fluorescence spectra change of WDFBT and WDFABT in buffer solution (10 μM) excited at 360 nm, respectively. Inset: emission intensity change of WDFBT and WDFABT induced by different equiv. of Hg^2+^ ions, and fluorescent color change of WDFBT and WDFABT upon addition of Hg^2+^ ions.

Realization aqueous environment detection of Hg^2+^ is essential for practical application. Here we also studied the detection ability of WDFBT and WDFABT towards Hg^2+^ in water solution. As shown in Fig. 3, both WDFBT and WDFABT shown good detection to Hg^2+^ in aqueous environment with the similar spectra changes as their compounding organic soluble dyes. And WDFABT also shown more sensitive toward Hg^2+^ than WDFBT.

### Selectivity for Hg^2+^

**Fig. 4:**
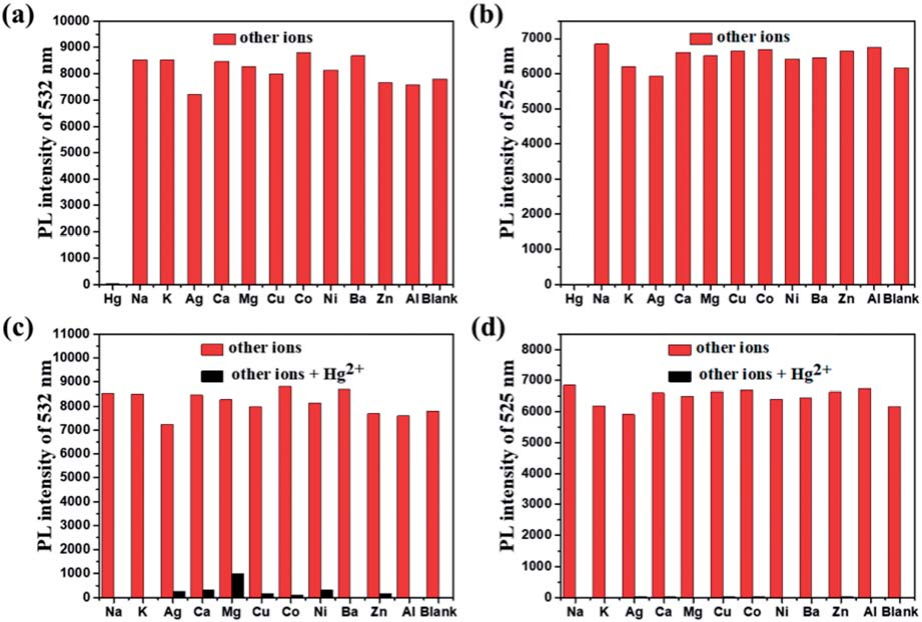
Relative fluorescence intensity of 10 μM DFBT (a) and (c) and DFABT (b) and (d) in CH_2_Cl_2_ upon excitation at 360 nm (the isosbestic point). Red bars represent the addition of 10 equiv. of indicated metal ions. Black bars represent the addition of 5 equiv. of Hg^2+^ together with 10 equiv. of the indicated metal ions.

For an excellent probe, high selectivity also is a matter of necessity. Herein, the selective coordination studies of probe DFBT and DFABT by fluorescent spectroscopy were then extended to different metal ions including Ba^2+^, Ag^+^, Ca^2+^, Co^2+^, Cr^3+^, Cu^2+^, K^+^, Na^+^, Mg^2+^, Al^3+^, Ni^2+^ and Zn^2+^. As shown in Fig. 4(a) and (b), only addition of Hg^2+^ ions resulted in prominent complete fluorescence quenching, whereas quite slight variations of fluorescence spectra were observed upon addition of the equal amount of the other metal ions, which shown a much more obvious response compared with other metal ions. Therefore, DFBT and DFABT as a sensor for Hg^2+^ ions could achieve high selectivity over other metal ions. To further validated the high selectivity of DFBT and DFABT for the detection of Hg^2+^ ions, the competitive experiments were carried out in the presence of Hg^2+^ ions mixed with other metal ions. As shown in Fig. 4(c) and (d), there were slight change in the emission intensity and colorimetric photographs by comparison with or without other metal ions, which indicated that the selectivity of DFBT and DFABT to Hg^2+^ ions was hardly affected by these commonly coexistent metal ions.

**Fig. 5:**
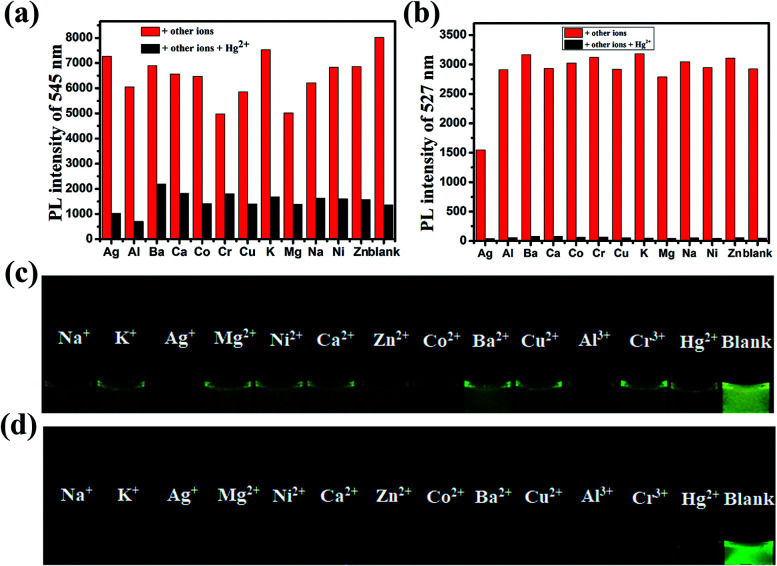
Competitively fluorescent response tests of WDFBT and WDFABT to various metal ions in buffer solution (10 μM): (a) and (b) emission intensity change of WDFBT and WDFABT, respectively. The red bars represent the WDFBT and WDFABT buffer solution (10 μM) mixed with various other metal ions; the black bars represent the WDFBT and WDFABT buffer solution (10 μM) mixed with Hg^2+^ ions and other metal ions. (c) and (d) image of the fluorescence color change of WDFBT and WDFABT buffer solution mixed with Hg^2+^ ions and other metal ions, respectively. (WDFBT buffer solution mixed with 300 equiv. Hg^2+^ ions and same equiv. other metal ions, WDFABT buffer solution mixed with 150 equiv. Hg^2+^ ions and same equiv. other metal ions).

The selectivity of WDFBT and WDFABT towards Hg^2+^ in water solution were also studied. As shown in Fig. 5, these two dyes both exhibited good selectivity and anti-interference ability. Especially, WDFABT shown better detection performance than WDFBT with obvious fluorescent changes even under the present of other ions.

### Binding constant (*K*) and binding ratio for Hg^2+^

**Fig. 6:**
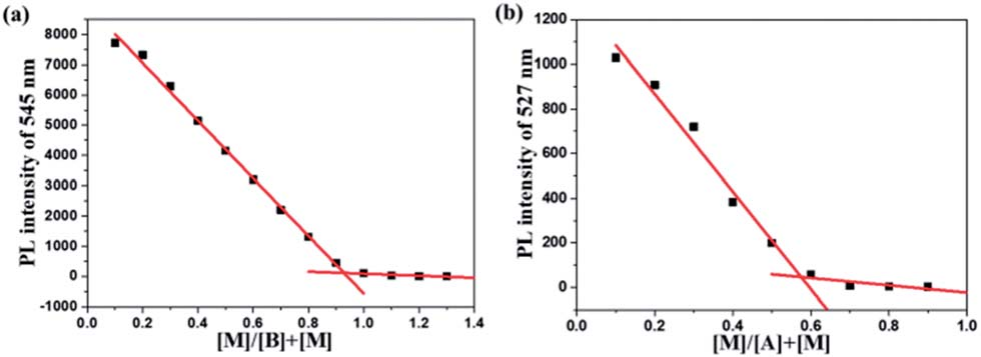
Job plot for WDFBT (a) and WDFABT (b) with Hg^2+^ ions in buffer solution.

According to the fluorescence titration experiment of the two dyes for Hg^2+^ ions, the binding constant (*K*) of DFBT and DFABT were determined to be 2.33 × 10^4^ M^−1^ and 8.38 × 10^5^ M^−1^ respectively. The detection limit of DFBT and DFABT as an “on–off-type” fluorescent probe for the analysis of Hg^2+^ ions was 10^−6^ M and 10^−7^ M respectively, which is at the same level as the similar reported probes.^36–38^ A Job's plot analysis was also carried out to further demonstrate the binding ratio between the probes and Hg^2+^ and the specific measuring methods were described in ESI.†^31,34^ The result of Job plot analysis (Fig. 6 (a) and (b)) confirmed a 2 : 1 binding ratio between WDFBT and Hg^2+^and a 1 : 1 binding ratio between WDFABT and Hg^2+^.

### Detection mechanism

**Fig. 7:**
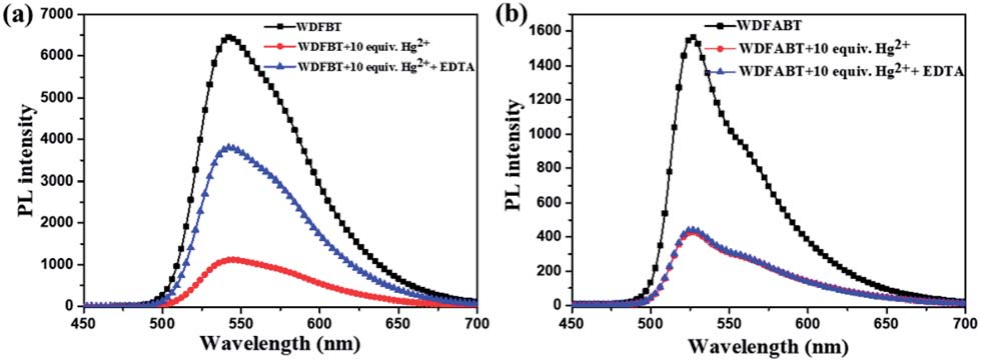
Changes in the emission spectra of probes in buffer solution before and after adding Hg^2+^ and EDTA, respectively: (a) WDFBT, (b) WDFABT.

From the above experimental results, we can see that these two fluorescent probes show different detection performance towards Hg^2+^ ions. To further reveal the background chemical process during the detection, detailed studies on the detection mechanism of these two kinds of fluorescent probes has been carried out. Firstly, the reversibility of these two kinds of probes have been studied *via* checking the repeatability of the fluorescent signal following the addition of ethylenediaminetetraacetic acid (EDTA), a kind of strong chelate for Hg^2+^ ions,^22,31^ into the detection solutions. As shown in Fig. 7(a) and (b), an obvious fluorescence quenching appeared upon addition of Hg^2+^ ions to WDFBT and WDFABT aqueous solution. When excess EDTA was added subsequently into the mixed solution, however, the two florescent probes showed different emission recovery phenomenon. Compared to the obvious emission recovering of WDFBT there was almost no emission recovery for WDFABT, which was probably because that the chemical structure of WDFABT has been broken in the presence of Hg^2+^ ions as the result of hydration reaction of acetylene group catalysed by mercury ions catalytic, also known as the Kucherov reaction.

To further demonstrate this assumption, and considering the good solubility in organic solvent, here we have chosen DFBT and DFABT to study the structure changes before and after adding Hg^2+^ ions and EDTA utilizing mass spectrometry. As shown in Fig. S16,† when Hg^2+^ ions were added, both the original peak of DFBT and DFABT disappeared (calcd 976.47 for DFBT and 1024.47 for DFABT), meanwhile a new peak appeared at *m*/*z* 751.469 for DFBT which corresponding to the segment of DFBT subtracting the *tert*-butyl ester group, and a series of fragment peaks appeared near *m*/*z* 555.919 for DFABT which probably attributed to the decomposition of DFABT. Subsequently, excess EDTA was added, a new peak at *m*/*z* 953.915 was appeared for DFBT, of which 24 protons in *t*-butyl ester group was interrupted (calcd 976.47 for DFBT), whereas there still was a series of fragment peaks near *m*/*z* 540.202 for DFABT. Hence, the results of MS data indicated that the conjugation framework of DFBT still existed but that of DFABT was destroyed in the presence of Hg^2+^ ions. A Job's plot analysis was also carried out to demonstrate the binding ratio between the probes and Hg^2+^ ions and the specific measuring methods were described in ESI.†^31,34^ The result of Job plot analysis (Fig. 6) confirmed a 2 : 1 binding ratio between WDFBT and Hg^2+^ ions and a 1 : 1 binding ratio between WDFABT and Hg^2+^ ion.

**Fig. 8:**
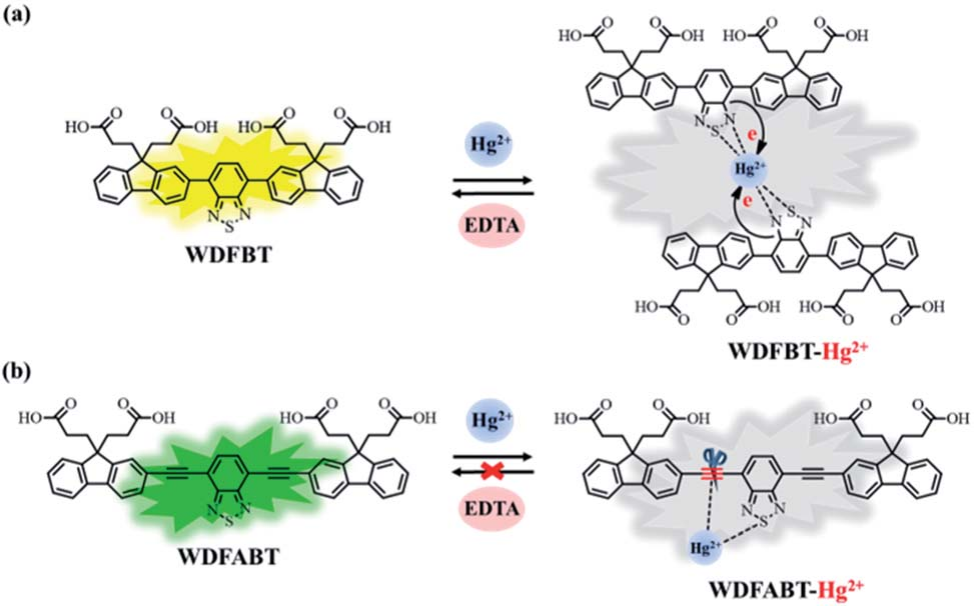
The possible detection mechanism of probes for Hg^2+^ ions: (a) WDFBT, (b) WDFABT.

Based on the PL spectra and mass spectra data, we concluded that, for probe DFBT and WDFBT, there was a reversible coordination reaction with a 2 : 1 binding ratio between probe and Hg^2+^ ions, which caused the fluorescence quenching *via* the excited-state charge transfer from electron-rich BT moiety to electron-deficient Hg^2+^ ions as shown in Fig. 8(a).^34,35^ And, for DFABT and WDFABT, there was an irreversible decomposition reaction with a 1 : 1 binding ratio between probe and Hg^2+^ ions, which caused fluorescence quenching mainly because of the conjugation framework destruction of probe molecule as shown in Fig. 8(b).

## Conclusions

In summary, we have successfully developed two kinds of fluorescent probe for Hg^2+^ ions based on the carboxyl-functionalized fluorine trimer with a benzo[2,1,3]thiadiazole bridge as a binding site. And the detection of Hg^2+^ in organic and aqueous environment were realized respectively. By changing the bridged modes between the functional group from C–C single bond to C≡C triple bond, different type fluorescent probes with different binding ability to Hg^2+^ ions were realized. Compared with dyes bridged with C–C single bond, dyes bridged with C≡C triple bond exhibited higher sensitivity due to the synergetic coordination effect of the triple bond and sulfur atom to Hg^2+^ ions. Both of two kinds probes shown a great selectivity over other competitive ions. However, the detection limit of WDFBT (1.08 mM) and WDFABT (0.29 mM) for Hg^2+^ ions in aqueous environment was still low, and the future work will focus on how to promote the detection limit of this type probes and realize the stable detection for Hg^2+^ ions *in vitro* and *vivo* through the molecular structure modification.

## Conflicts of interest

The authors declare that they have no conflict of interest.

## Supplementary Material

RA-008-C8RA00295A-s001
