# Supplementary material for: Rational design of fluorescent probe for Hg2+ by changing the chemical bond type
Source: RSC Adv. 2018 Mar 29;8(22):12276–81. doi: 10.1039/c8ra00295a (PMC9079269; doi:10.1039/c8ra00295a)
Supplement: RA-008-C8RA00295A-s001 [file RA-008-C8RA00295A-s001.pdf]

Electronic Supplementary Material (ESI) for RSC Advances.
This journal is © The Royal Society of Chemistry 2018

Supporting Information

Rational Design of Fluorescent Probe for Hg^{2+} by Changing the Chemical Bond Type

Determination of the binding constant

Job's method

Determination of relative fluorescent quantum yields

Synthetic experimental

^{1}H NMR, ^{13}C NMR and Mass spectra

Competitively fluorescent response tests of WDFBT and WDFABT

Reversibility tests of DFBT and DFABT

References

Determination of the binding constant

The binding constants of **DFBT** and **DFABT** were determined from PL spectroscopic titrations in the buffer aqueous solution according to the equation as described by Zhao:[1]

$I_F^0/(I_F-I_F^0) = [a/(a-b)][1/K[M]+1]$

I_F^0 = emission intensity of **DFBT** and **DFABT** at 547 nm and 525 nm, respectively.

I_F = emission intensity of **DFBT** and **DFABT** at 547 nm and 525 nm upon the addition of different equivalents of Hg^{2+} ions.

[M] = concentration of Hg^{2+} ions.

a, b = constant.

The binding constant K is given by the ratio intercept/slope.

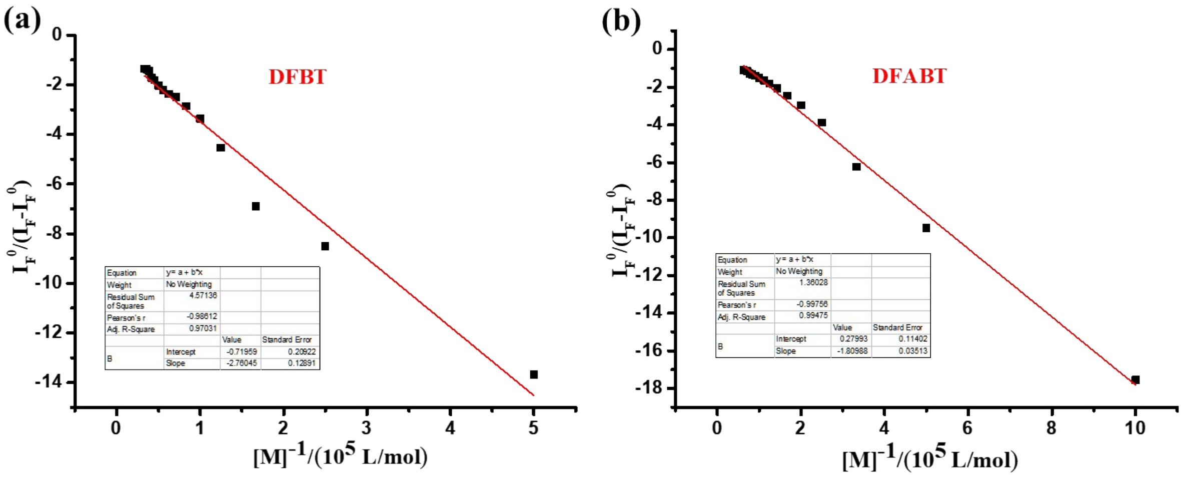

Job’s plot measurement

Job’s plot used to further identify the stoichiometry of host-gust complex. The two dyes and Hg^{2+} ions were dissolved in the buffer solution of citric acid and Na_2HPO_4 and deionized water respectively at an apposite concentration, and then mixed and diluted the dyes solution and Hg^{2+} ions solution to control the concentrations. The sum of the total concentration of **WDFBT** (**WDFABT**) and Hg^{2+} namely [B]+[M] ([A]+[M]) was maintained to be constant. Meanwhile, the ratio of [B]+[M] was varied from 1:9 to 9:1 and then the mercury ions solution was added continuously until the fluorescent intensity of **WDFBT (WDFABT)** unchanged, moreover, the ratio of [A]+[M] was vary from 1:9 to 9:1. The PL intensity values were plotted against the mole fraction of the Hg^{2+} after recording the emission spectra of above solutions.

Determination of relative fluorescent quantum yield

The relative fluorescent quantum yield of dyes were determined from UV-vis spectra and fluorescent spectra by using rhodamine-B as a reference, and then calculated according to the equation as described below:

Φ(x) =(Φ(s)*A(s)*F(s)/(A(x)*F(s)))*(η(x)/ η(s))^2;

Φ(s) = the fluorescent quantum yield of rhodamine-B in ethanol;

A(s) = the absorbance of rhodamine-B at 365 nm in ethanol;

F(s) = the integral area of emission spectra excited by 365 nm of rhodamine-B in ethanol;

A(x) = the absorbance of dyes at 365 nm;

F(x) = the integral area of emission spectra of dyes excited by 365 nm;

η(s) = the refractive index of ethanol solution;

η(x) = the refractive index of solvent;

wherein the maximum absorbance of above solutions was maintained in the range of 0.05 to 0.1, and the emission spectrum of corresponding solution was measured.

Synthetic experimental

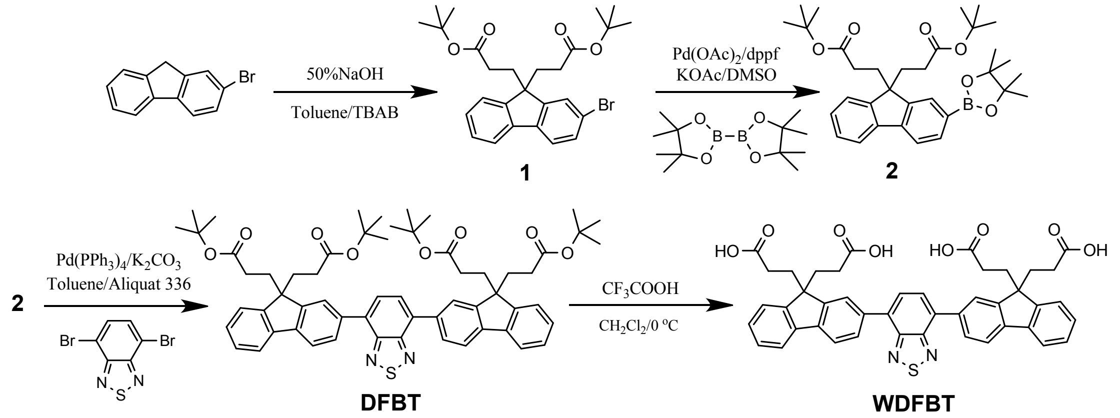

2-bromo-9,9-bis(3-(tert-butylpropanonate))fluorine (1)

2-bromofluorene (2.00 g, 8.16 mmol) and tetrabutylammonium bromide (100 mg, 0.03 mmol) were added to a two-necked round-bottom flask, and toluene (12 mL) were injected under an Ar atmosphere. After 60 min, 4.8 mL of solution 50 wt % aqueous NaOH was added dropwise, and the solution was stirred for 60 min. After that, tert-butyl acrylate (3.16 g, 24.64 mmol) was added dropwise, and the mixture was stirred at 25°C overnight. After completion of the reaction, the reaction product was extracted with dichloromethane, washed three times with HCl aqueous solution and deionized water, and dried with anhydrous $MgSO_4$. After the solvent was removed through rotational evaporation, the crude was purified by column chromatography (silica gel, petroleum ether/ dichloromethane, 10:1, v/v) to give 2.62 g of light yellow solid with a 64% yield. ^{1}H NMR (500 MHz, $CDCl_3$): δ = 7.66 (t, J=5.0Hz, 1H), 7.56 (d, J=5.0Hz, 1H), 7.50-7.47 (m, 2H), 7.37-7.34 (m, 3H), 2.35-2.30 (m, 4H), 1.49-1.45 (m, 4H), 1.31 (s, 18H).

2-(4,4,5,5-tetramethyl-1,3,2-dioxaborolan-2-yl)-9,9-bis(3-(t-butylpropanoate)) fluorene (2)

In a two-necked round-bottom flask, compound **1** (3.27 g, 6.5 mmol), $Pd(OAc)_2$ (43.8 mg, 0.195mmol), dppf (38.3 g, 0.39 mmol), KOAc (2.55 g, 26 mmol) and bis(pinacolato)diboron (2.1 g, 8.45 mmol) were dissolved in degassed DMSO under an Ar atmosphere. The mixture was stirred overnight at 90 ^{o}C. After cooling to room temperature, the reaction product was poured into water and extracted with dichloromethane three times. The organic layer was washed three times with water and then dried with anhydrous $MgSO_4$. After removing the solvent, the residue was purified by column chromatography (ethyl acetate/hexane, 1:4, v/v) to give compound **2** (2.4 g, 67 %) as a white solid. ^{1}H NMR (500 MHz, $CDCl_3$): δ = 7.83 (d, J=10.0Hz, 1H), 7.78 (s, 1H), 7.73-7.68 (m, 2H), 7.40-7.38 (m, 1H), 7.35-7.34 (m, 2H), 2.40-2.32 (m, 4H), 1.48-1.41 (m, 4H), 1.39 (s, 12H), 1.30 (s, 18H).

4,7-Bis(9,9-bis(3-(t-butylpropanoate))fluoren-2-yl)-2.1.3-benzothiadiazole (DFBT)

In a two-necked round-bottom flask, compound **2** (700 mg, 1.28 mmol) and 4, 7-dibromo-2.1.3-benzothiadiazole (117 mg, 0.40mmol), $Pd(PPh_3)_4$ (44 mg, 0.038 mmol) were added. After the reaction apparatus was degassed, the K_2CO_3 aqueous solution (2 M, 4 mL) and toluene (10 mL) were added under an Ar atmosphere. The mixture was heated at 90 ^{o}C with vigorous stirring for 12 hours and then cooled down to room temperature. The residue was extracted with CH_2Cl_2 and washed three times with water. Collecting the organic layer and dried with $MgSO_4$. The resulted

crude was purified with silica chromatography (dichloromethane/hexane, 3:2, v/v) to give compound **DFBT** as yellow-green powder (280 mg, 72%). ^{1}H NMR (500 MHz, $CDCl_3$): δ = 8.11 (d, J=10.0Hz, 2H), 7.98 (s, 2H), 7.92 (s, 2H), 7.89 (d, J=10.0Hz, 2H), 7.79 (d, J=10.0Hz, 2H), 7.44-7.37 (m, 6H), 2.50-2.38 (m, 8H), 1.70-1.56 (m, 8H), 1.31 (s, 36H). ^{13}C NMR (500 MHz, $CDCl_3$): δ =172.83, 154.26, 148.74, 148.60, 141.37, 140.78, 136.74, 133.33, 129.06, 128.08, 127.92, 127.67, 123.93, 123.22, 120.27, 120.02, 80.06, 53.77, 34.69, 30.23, 28.04 ppm. MS (MALDI-TOF) (m/z): 976.221.

4,7-Bis(9,9-bis(3''-propanoic))fluoren-2-yl)-2.1.3-benzothiadiazole (WDFBT)

In a 50 mL flask, compound **3** (50 mg, 0.051 mmol) was dissolved in CH_2Cl_2 (25 mL) under an Ar atmosphere and stirred at 0°C. After 40 min, trifluoroacetic acid (0.02 mL) was added dropwise and the mixture was stirred at room temperature overnight. After completion of the reaction, the mixture was dropped on basified water and the impurities were extracted with ethyl acetate. The aqueous layer was acidified and extracted with ethyl acetate, and the organic layer was washed with deionized water three times and collected. The solvent was removed to give **WDFBT** as a greenish yellow powder (30 mg, 78%). ^{1}H NMR (300 MHz, $(CD_3)_2SO$): δ = 11.87 (s, 4H), 8.15 (t, J=9.0Hz, 6H), 8.06 (d, J=9.0Hz, 2H), 7.96 (d, J=9.0Hz, 2H), 7.59 (d, J=9.0Hz, 2H), 7.47-7.38 (m, 4H), 2.45-2.33 (m, 8H), 1.58-1.41 (m, 8H), MS (MALDI-TOF) (m/z): 752.010. Elemental analysis calculated [%] for $C_{44}H_{36}N_2O_8S$: C 70.20, H 4.82, N 3.72; found C 65.63, H 4.87, N 3.27.

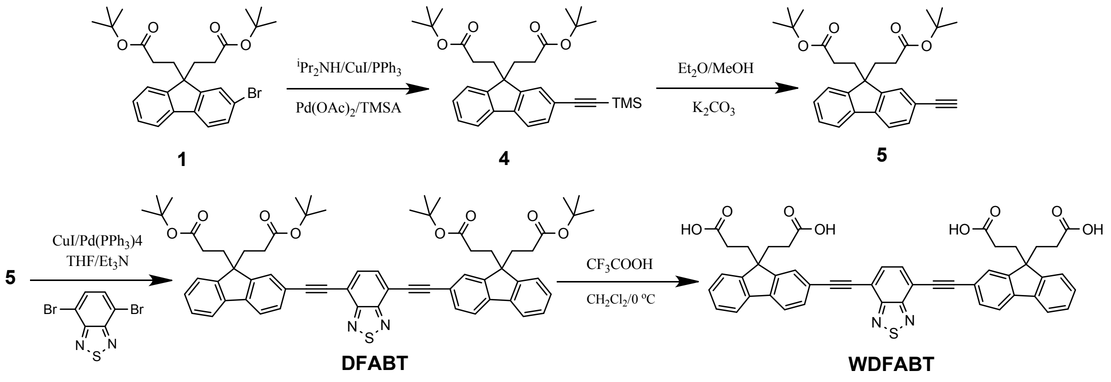

2-(2-(trimethylsilyl)ethynyl)-9,9-bis(3-(tert-butylpropanonate))fluorine (4).

In a two-necked round-bottom flask, compound **1** (2.00 g, 3.99 mmol), $Pd(OAc)_2$ (54.5 mg, 0.24 mmol), PPh_3 (109.2 mg, 0.42 mmol), CuI (53 mg, 0.28 mmol) were dissolved in diisopropylamine (16 mL) under an Ar atmosphere. Then, trimethylsilyl acetylene (2.77 g, 28.2 mmol) was injected dropwise, and the mixture was stirred at 70°C overnight. After completion of the reaction, the reaction solvent was removed, and then the residue was re-dissolved in CH_2Cl_2 and washed three times with deionized water. The resulted organic phase was dried with anhydrous $MgSO_4$.and filtered. The solvent was removed and the crude was purified in a chromatographic column (petroleum ether/dichloromethane, 50:1, v/v) to give compound **4** of light grey solid (1.6 g, 77%). ^{1}H NMR (500 MHz, $(CD_3)_2SO$): δ = 7.92-7.88 (m, 2H), 7.67 (s, 1H), 7.56 (d, J=5.0Hz, 2H), 7.43 (t, J=5.0Hz, 2H), 2.4-2.3 (m, 4H), 1.33 (t, J=10.0Hz, 4H), 1.29 (s, 18H), 0.3 (s, 9H).

2-ethynyl-9,9'-bis(3'-tert-butylpropanoate) fluorine (5)

In a 100 mL two-necked round-bottom flask, compound **4** (1.04 g, 2 mmol), K_2CO_3 (0.553 g, 4 mmol) was dissolved in a mixed solution of methanol (20 mL) and THF (20 mL), and the mixture was stirred at room temperature for 2 h. After that, the reaction solvent was evaporated under reduced pressure, the residue was re-dissolved in 50 mL dichloromethane, and washed three times with

deionized water. The organic phase was collected and dried with $MgSO_4$. The crude was purified by silica gel column chromatography (petroleum ethyl/acetate, 50:1, v/v) to give compound **5** as a white solid (0.784 g, 87%).^{1}H NMR (500 MHz, $(CD_3)_2SO$): δ = 7.90 (t, J=7.5 Hz, 2H), 7.68 (s, 1H), 7.56-7.52 (m, 2H), 7.43 (d, J=10.0Hz, 2H), 4.26 (s, 1H), 2.34 (t, J=7.5Hz, 4H), 1.34 (t, J=7.5Hz, 4H), 1.28 (s, 18H).

4,7-Bis(9,9′-bis(3′-tert-butylpropanoate))fluoren-2-ethynyl)-2.1.3-benzothiadiazole (DFABT)

In a 50 mL two-necked round-bottom flask, compound **5** (669 mg, 1.5 mmol), 4,7-dibromo-2.1.3-benzothiadiazole (146 mg, 0.50mmol), $Pd(PPh_3)_4$ (104 mg, 0.09 mmol), CuI (17 mg, 0.09 mmol) were dissolved in a mixed solution of THF (5 mL) and triethylamine (TEA) (5 mL) under an Ar atmosphere. After degassing, the mixture was stirred at 80°C for 24 h. After the mixture was cooled to room temperature, the reaction solvent was removed, and the residue was re-dissolved in dichloromethane, washed three times, and dried with anhydrous $MgSO_4$. After that, the crude was purified by silica gel column chromatography (petroleum ethyl/acetate, 20:1, v/v) to give the compound **DFABT** as an orange solid (350 mg, 68%). ^{1}H NMR (300 MHz, $CDCl_3$): δ = 7.85 (s, 2H), 7.75-7.69 (m, 8H), 7.43-7.36(m, 6H), 2.39 (t, J=7.5Hz, 8H), 1.52 (t, J=7.5Hz, 8H), 1.32 (s, 36H). ^{13}C NMR (500 MHz, $CDCl_3$): δ =172.58, 154.44, 148.64, 148.43, 142.22, 140.34, 132.51, 131.88, 128.43, 127.78, 126.52, 123.21, 121.36, 120.49, 120.09, 117.22, 98.38, 85.94, 80.20, 77.29, 77.04, 76.78, 53.68, 34.65, 30.03, 28.04 ppm. MS (MALDI-TOF) (m/z): 1023.763.

4,7-Bis(9,9-bis(3''-propanoic))fluoren-2-ethynyl)-2.1.3-benzothiadiazole (WDFABT)

In a 50 mL two-necked round-bottom flask, compound **6** (103 mg, 0.1 mmol) was dissolved in dichloromethane (10 mL) under a Ar atmosphere and the mixture was stirred for 60 min at 0°C. After that, trifluoroacetic acid (0.03 mL) was added dropwise and the mixed solution was stirred at room temperature overnight. After completion of the reaction, the subsequent treatment of the reaction product was the same as compound **WDFBT**. After removing the solvent, **WDFABT** was obtained as a deep orange powder (60 mg, 75%). ^{1}H NMR (500 MHz, $(CD_3)_2SO$): δ = 11.90 (s, 4H), 8.05 (s, 2H), 8.02 (d, J=10.0Hz, 2H), 7.97 (d, J=10.0Hz, 2H), 7.87 (s, 2H), 7.73 (d, J=10.0Hz, 2H), 7.60 (d, J=5.0Hz, 2H), 7.47-7.45 (m, 4H), 2.53-2.39 (m, 8H), 1.42-1.39 (m, 8H). MS (MALDI-TOF) (m/z): 798.230. Elemental analysis calculated [%] for $C_{48}H_{36}N_2O_8S$: C 71.99, H 4.53, N 3.50; found C 67.02, H 4.96, N 3.34.

^{1}H NMR, ^{13}C NMR and Mass spectra

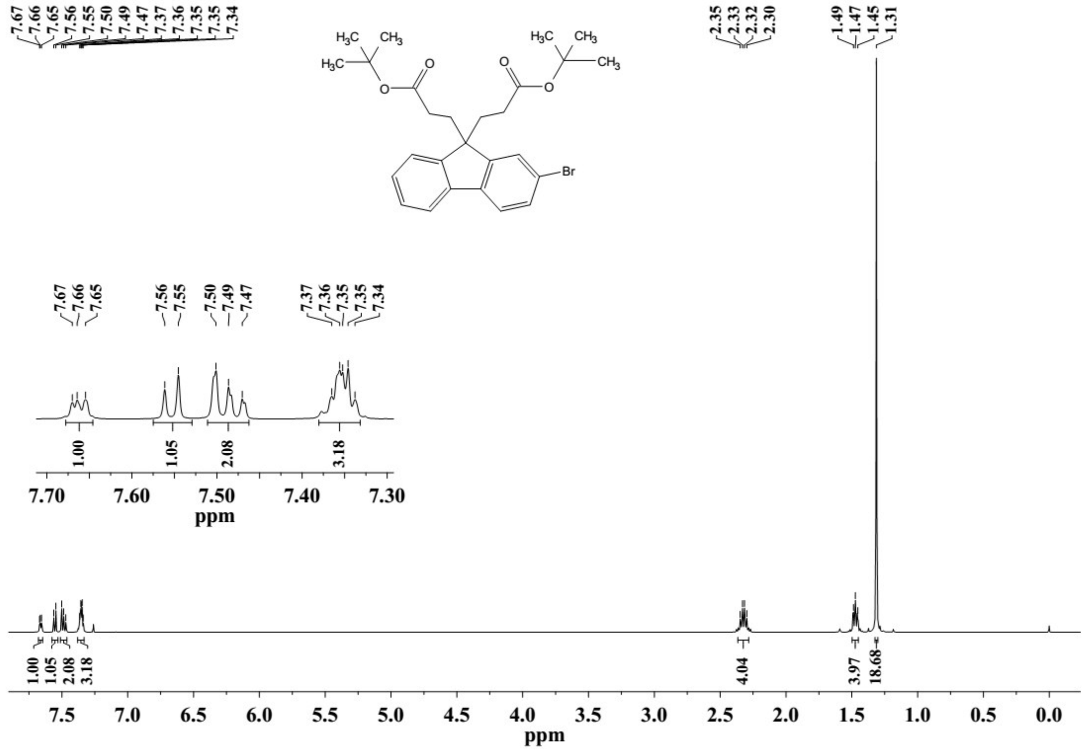

Figure S1. ^{1}H NMR spectrum of compound 1.

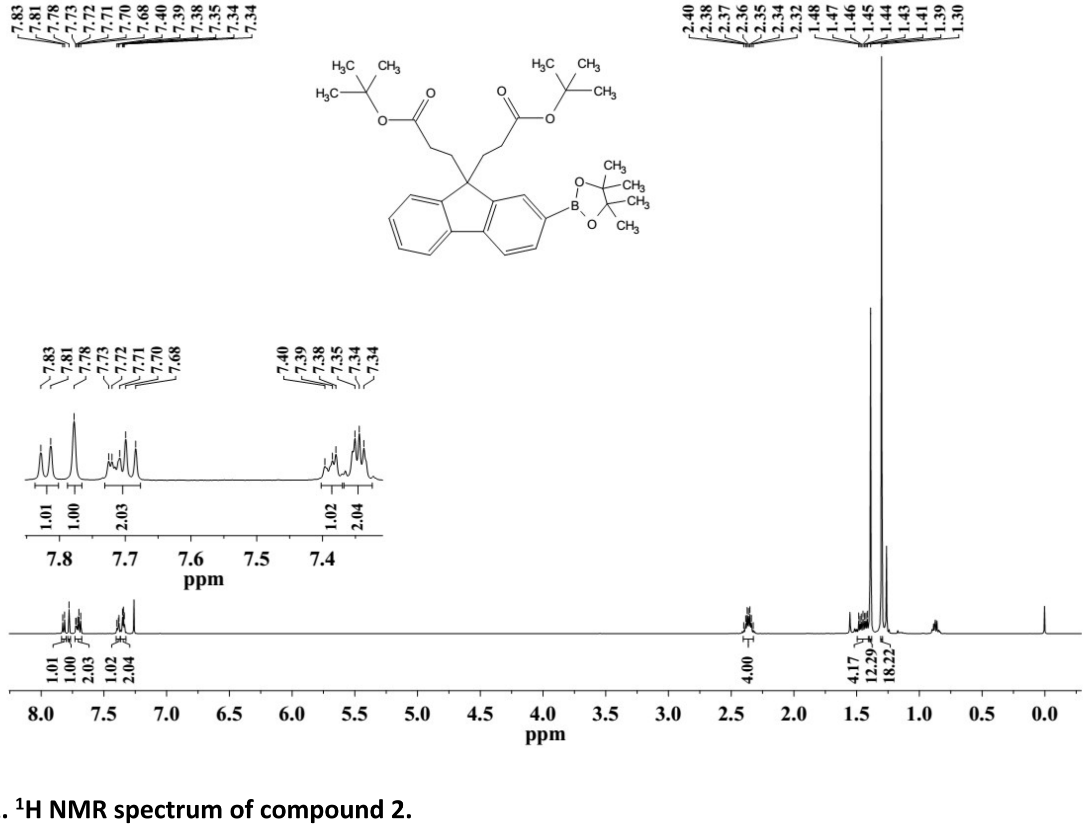

Figure S2. ^{1}H NMR spectrum of compound 2.

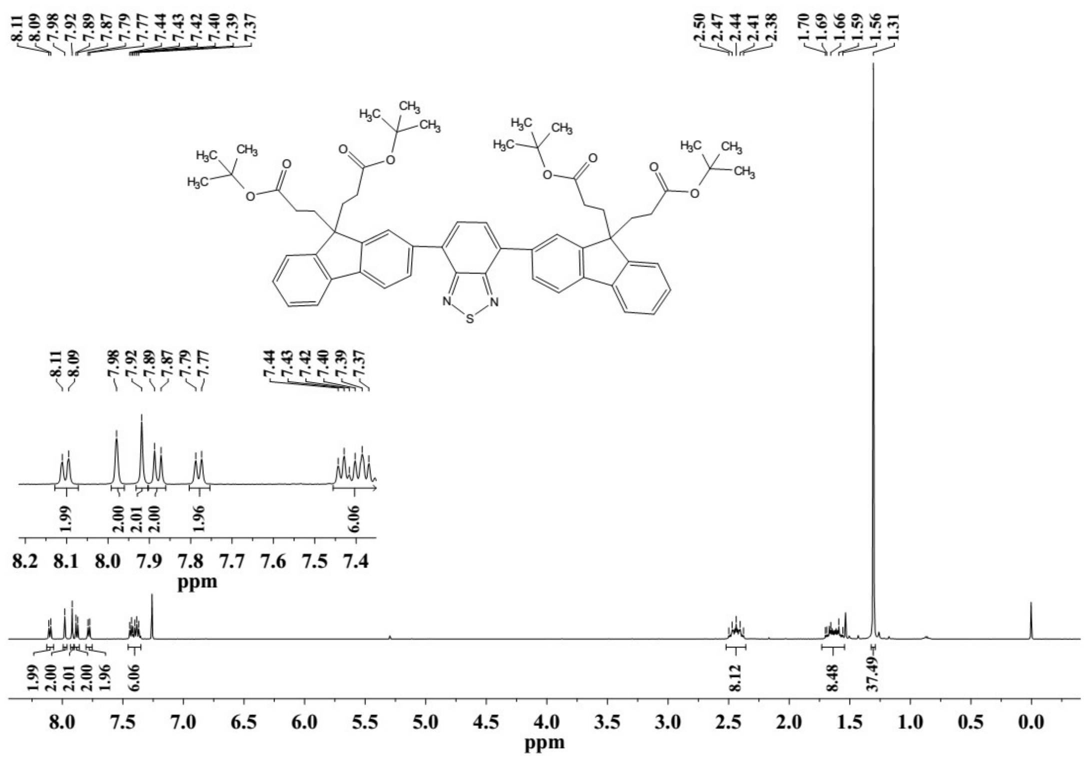

Figure S3. ^{1}**H NMR spectrum of compound DFBT.**

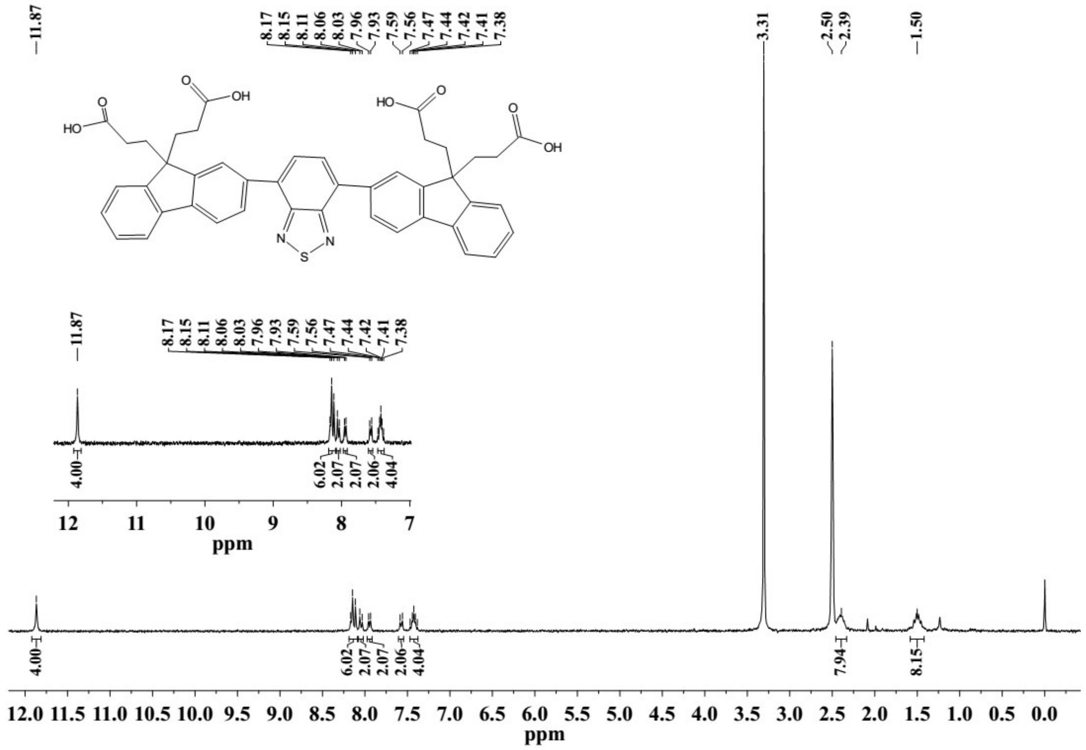

Figure S4. ^{1}**H NMR spectrum of WDFBT.**

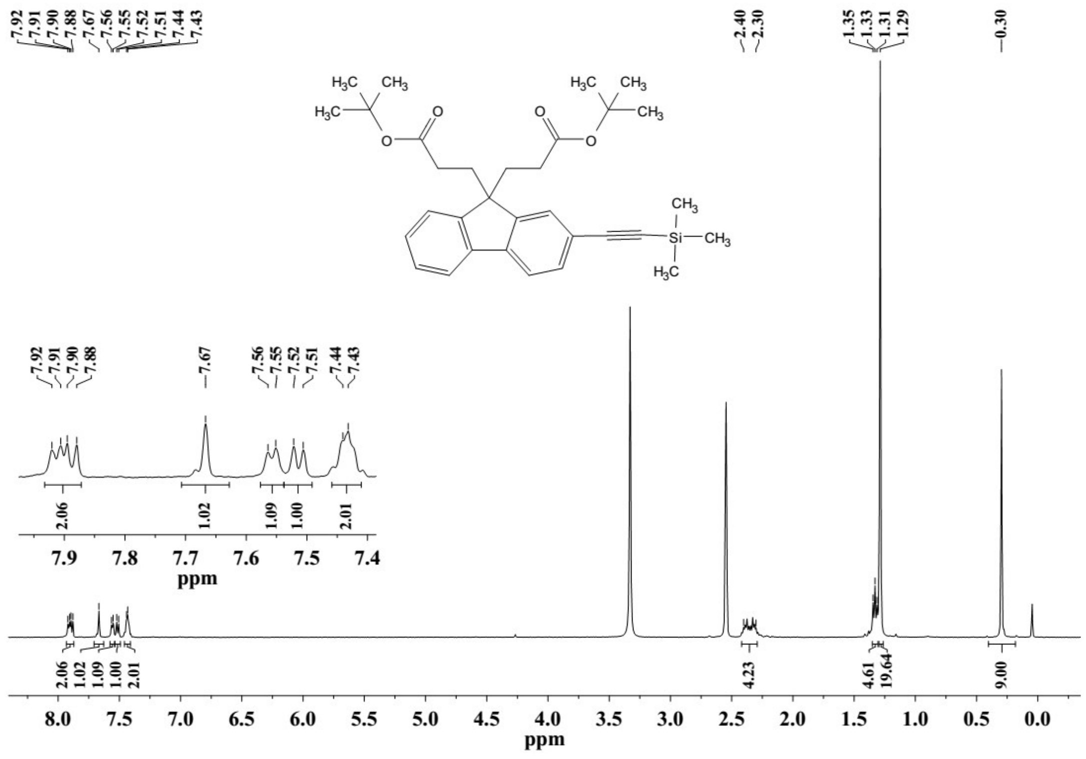

Figure S5. ^{1}**H NMR spectrum of compound 4.**

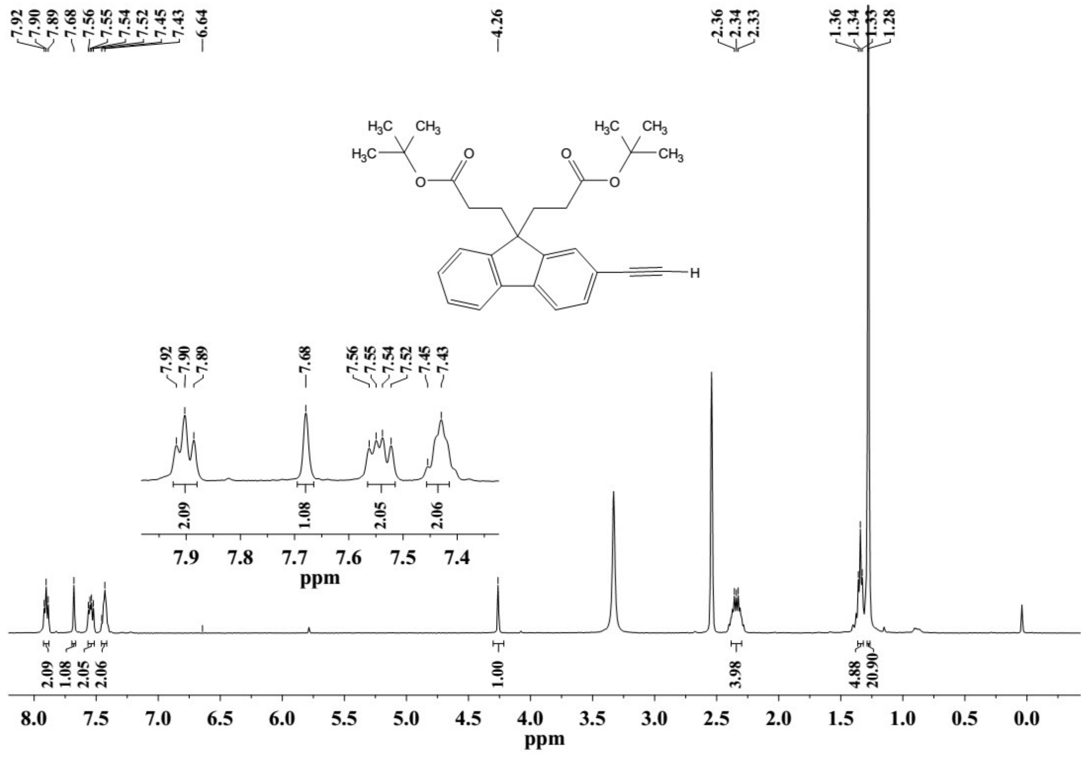

Figure S6. ^{1}**H NMR spectrum of compound 5.**

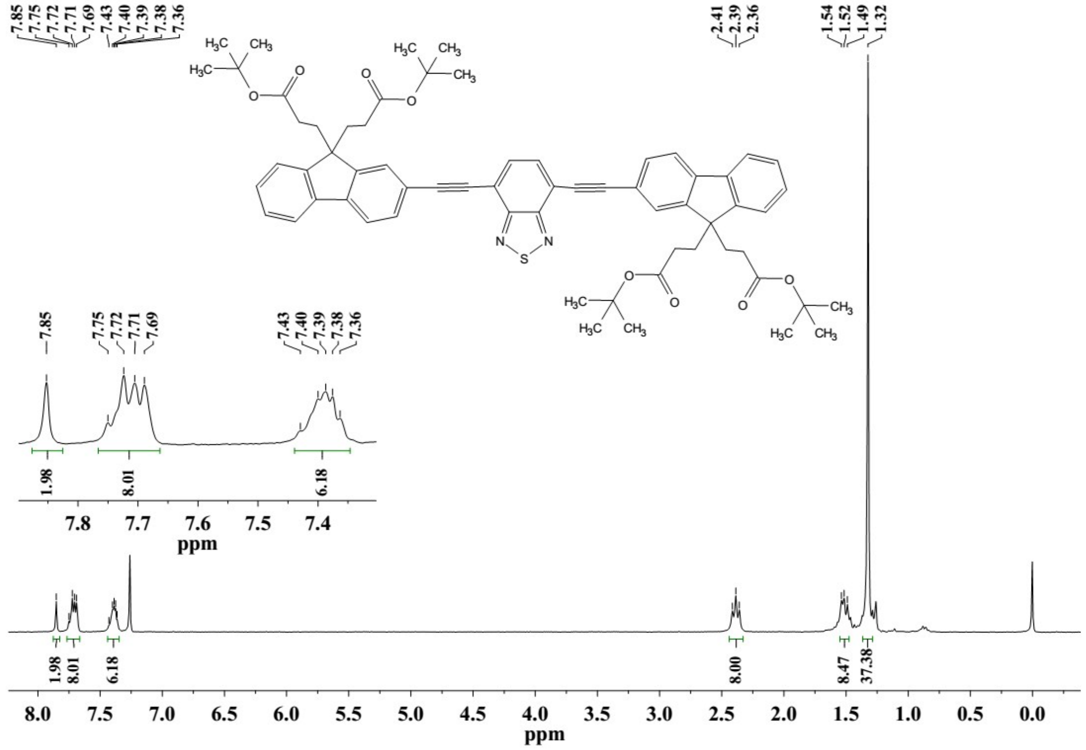

Figure S7. ^{1}H NMR spectrum of compound DFABT.

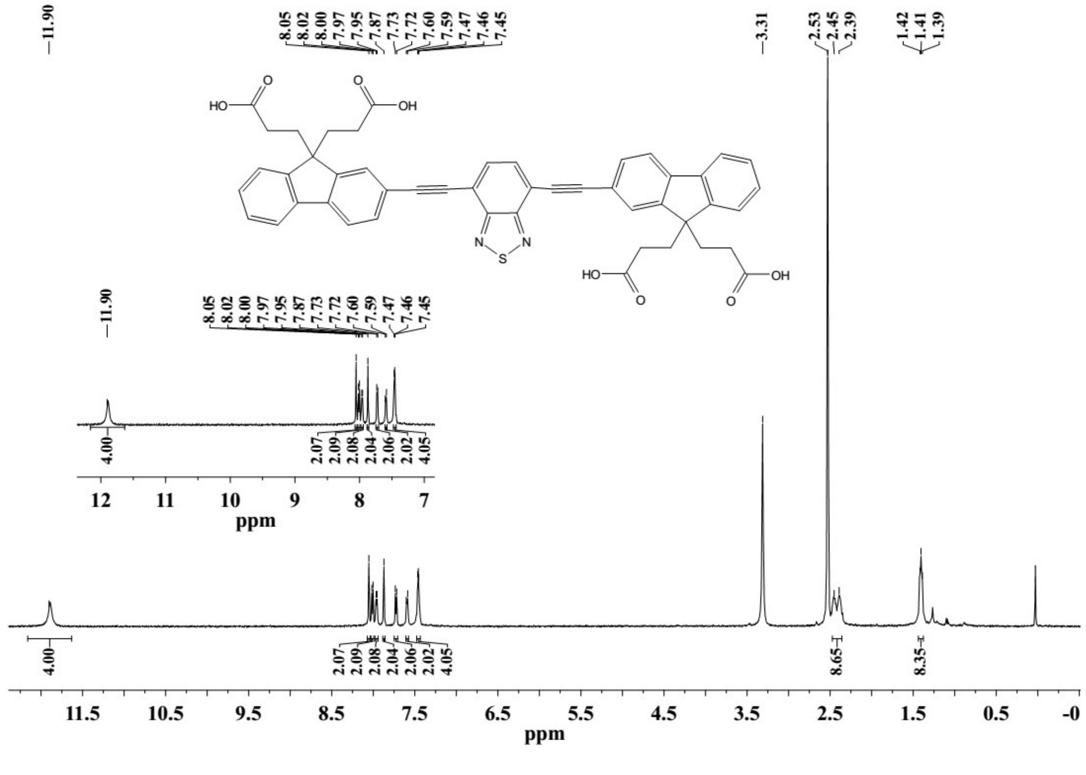

Figure S8. ^{1}H NMR spectrum of WDFABT.

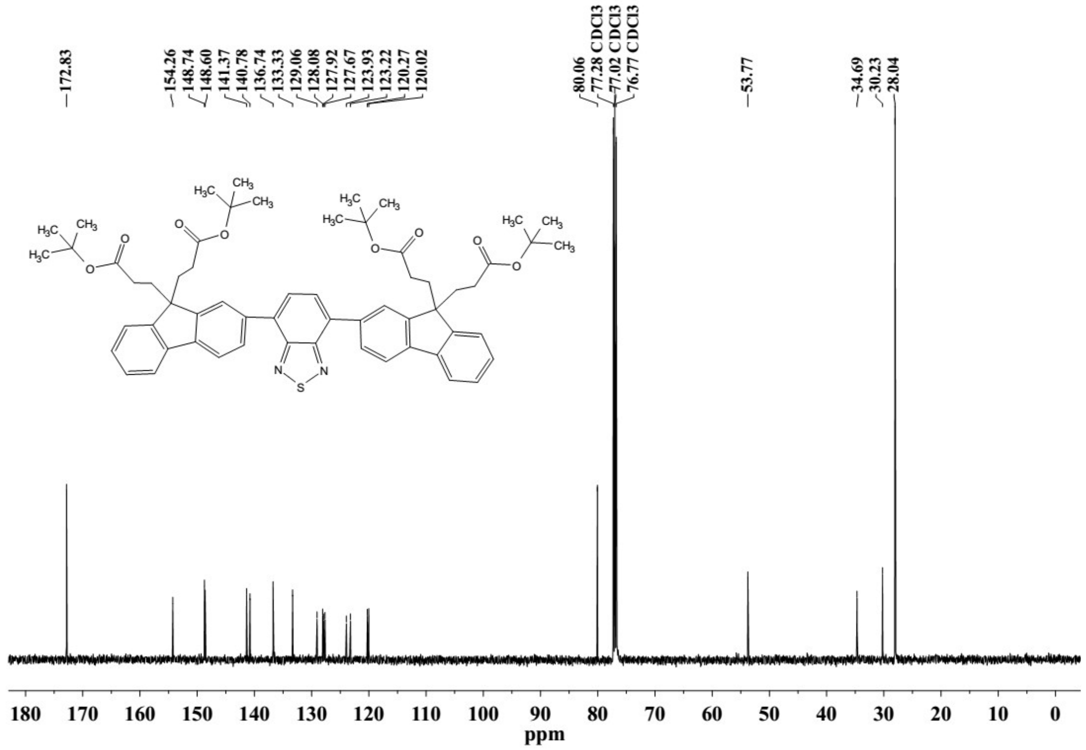

Figure S9. ^{13}C NMR spectrum of compound DFBT.

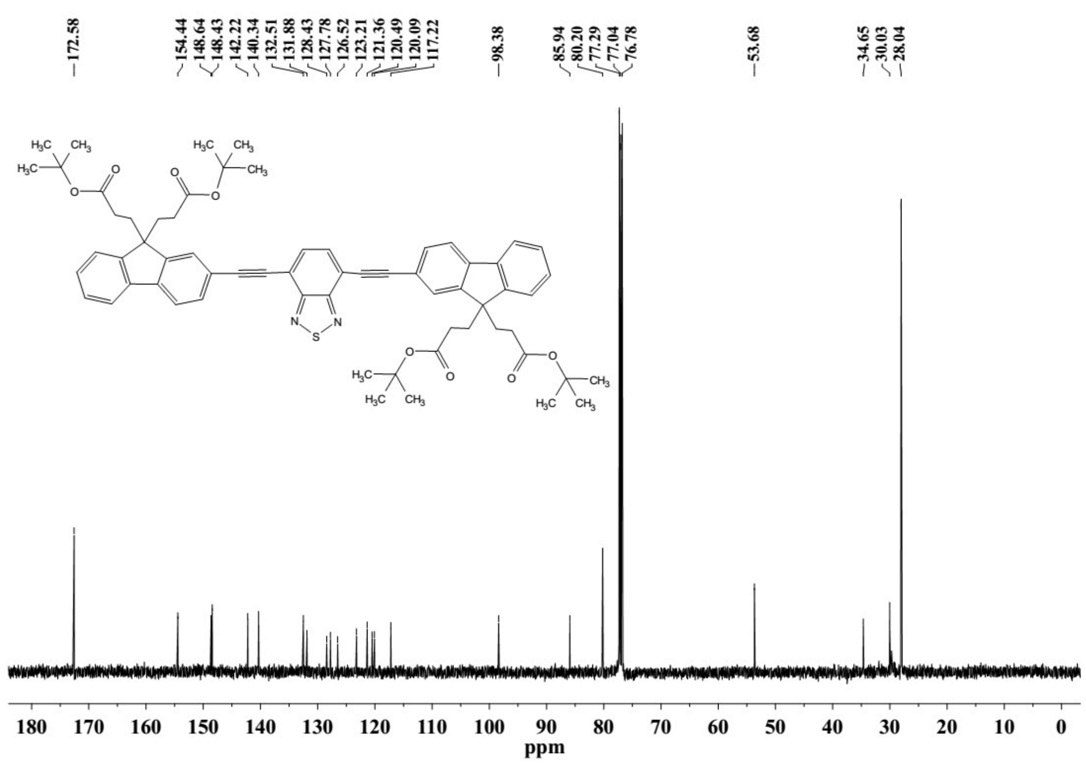

Figure S10. ^{13}C NMR spectrum of compound DFABT.

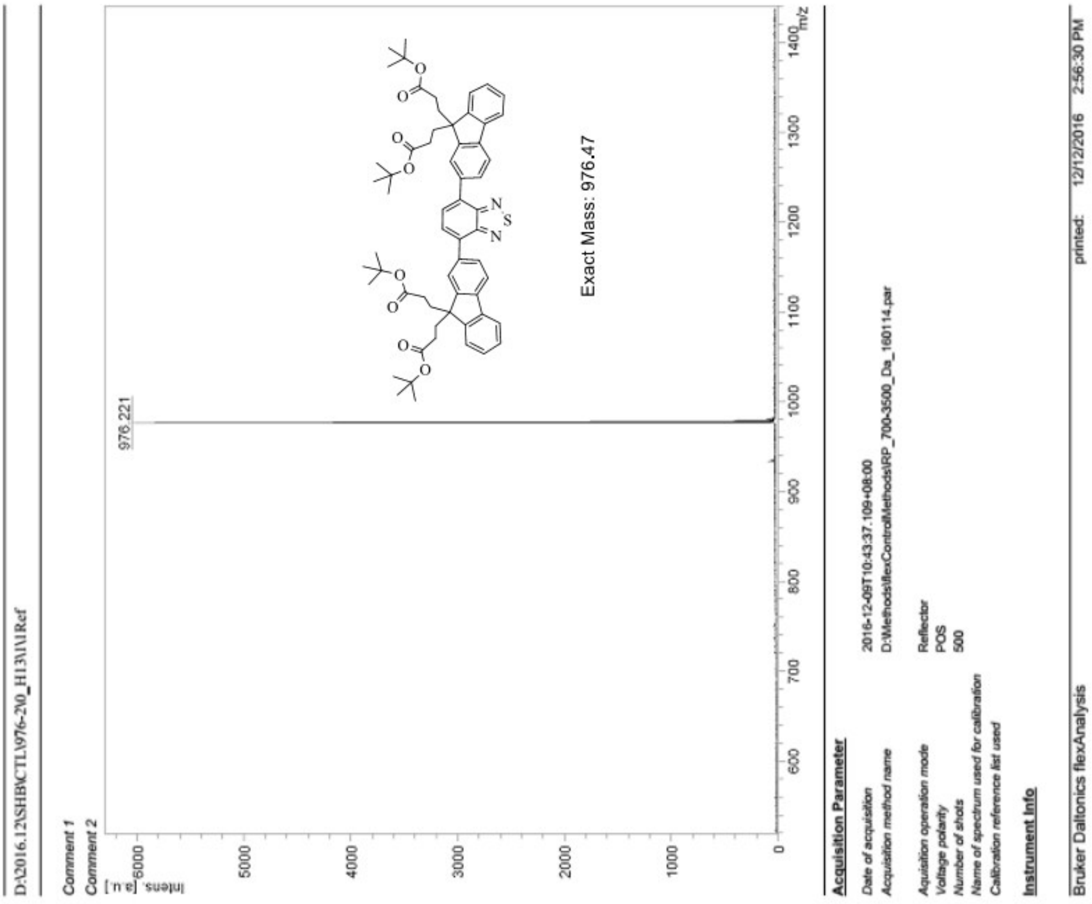

Figure S11. Mass spectra of compound DFBT.

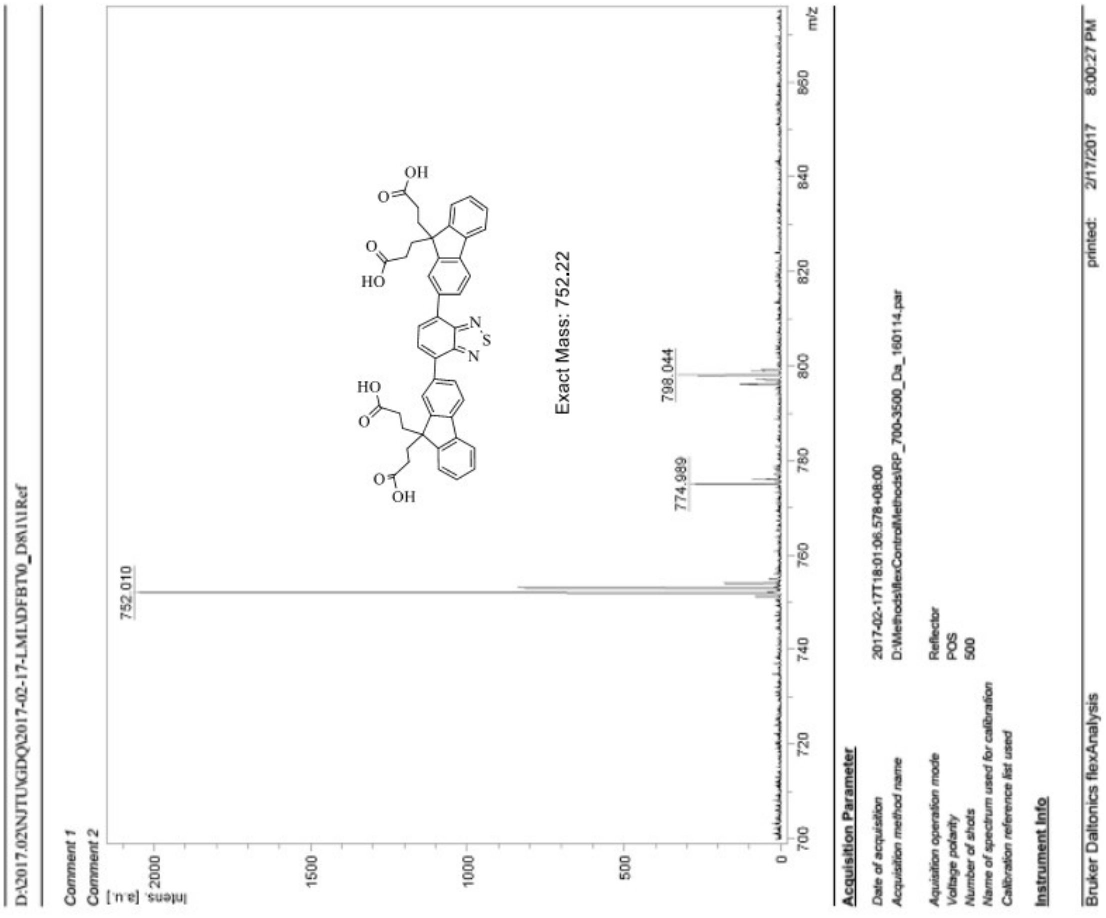

Figure S12. Mass spectra of WDFBT.

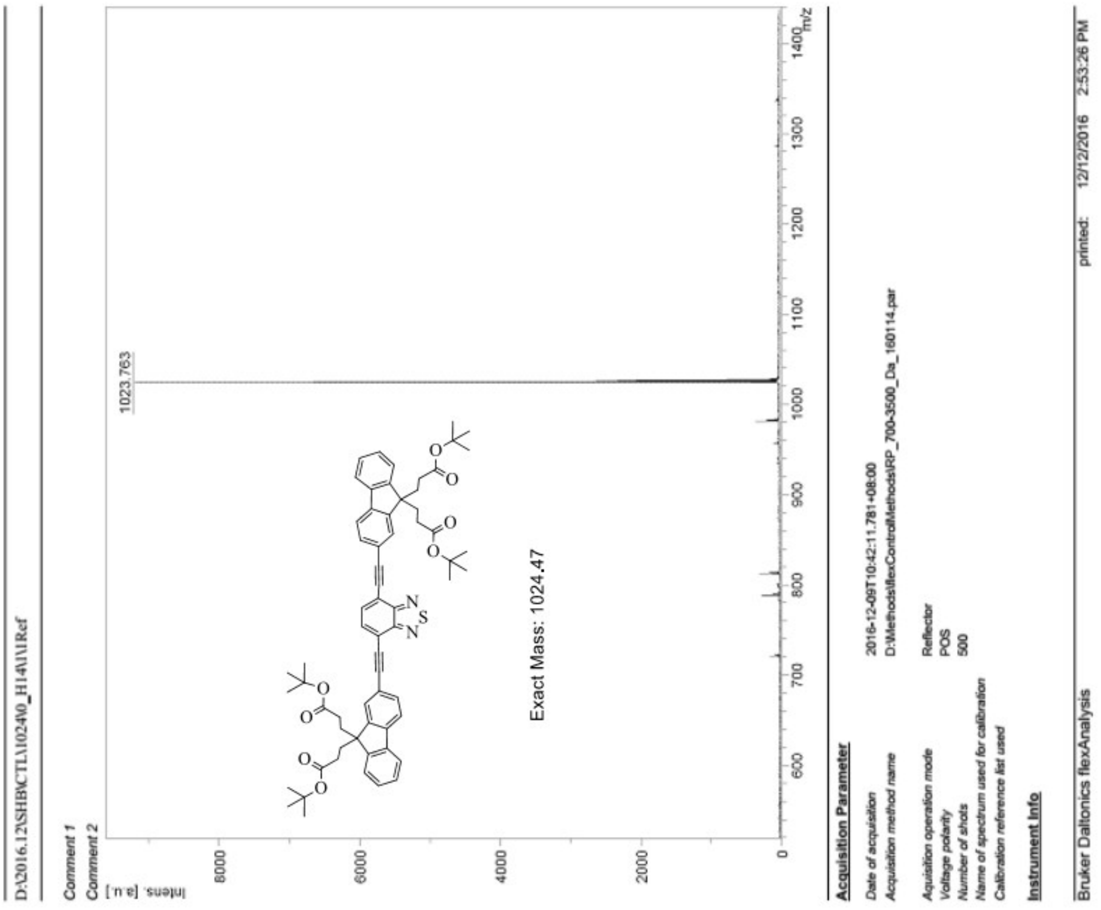

Figure S13. Mass spectra of compound DFABT.

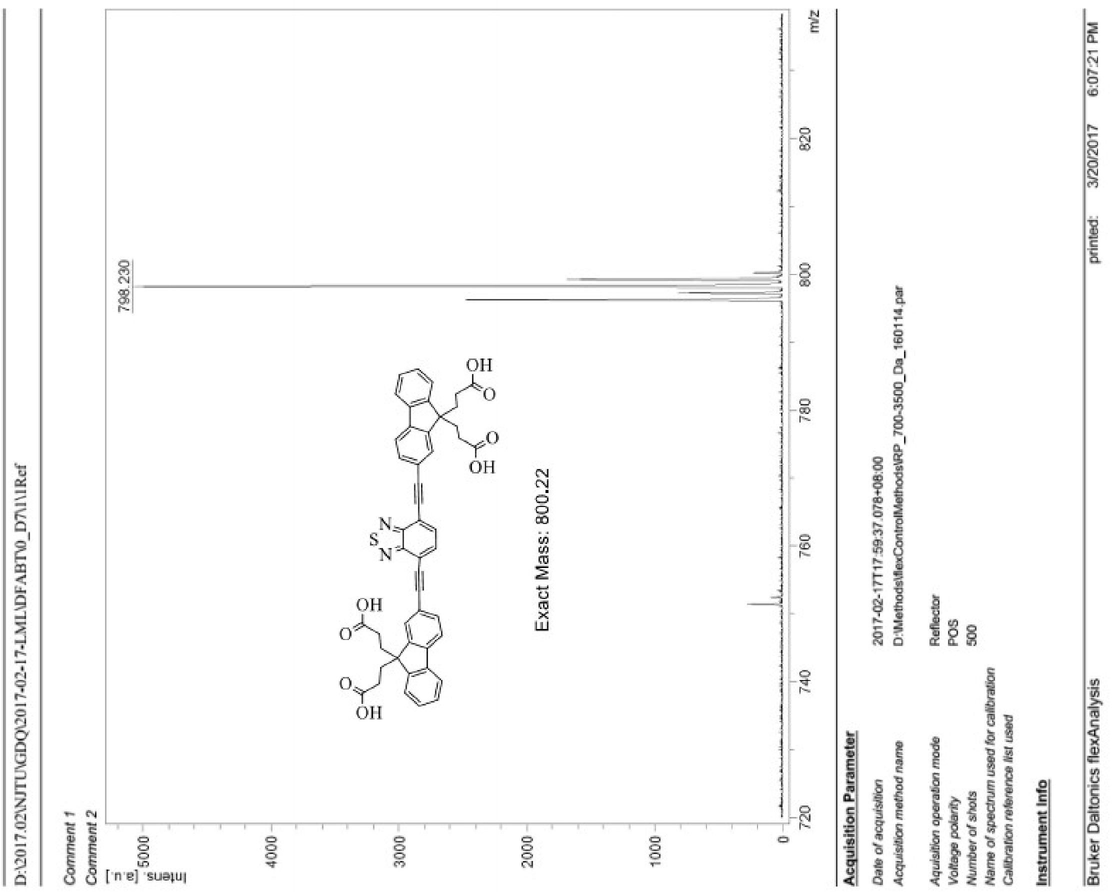

Figure S14. Mass spectra of WDFABT.

Competitively fluorescent response tests of WDFBT and WDFABT

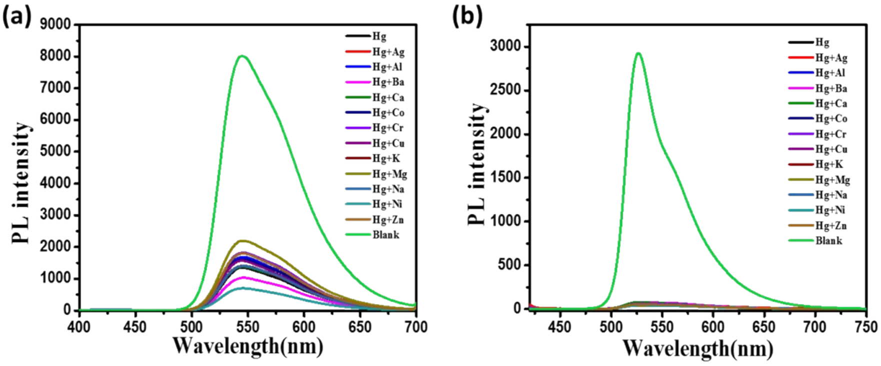

Figure S15. Emission spectras of WDFBT and WDFABT buffer solution (10 μM) mixed with equivalent Hg^{2+} ions and other metal ions: (a) WDFBT, (b) WDFABT.

Reversibility tests of DFBT and DFABT

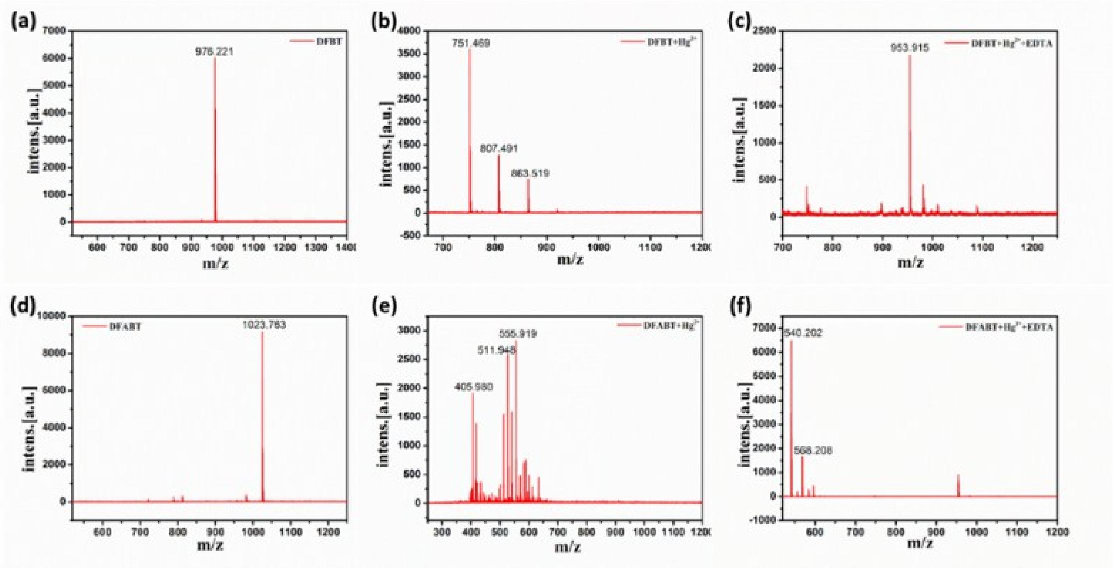

Figure S16. Change in the mass spectra of DFBT and DFABT in CH_2Cl_2 solution before and after adding Hg^{2+} and EDTA, respectively: (a)-(c) DFBT, (d)-(f) DFABT.

References

1. Zhao, Q.; Cao, T.; Li, F.; Li, X.; Jing, H.; Yi, T.; Huang, C. *Organometallics* 2007, **26**, 2077-2081.
